# Meta-analysis of metabolome QTLs in *Arabidopsis*: trying to estimate the network size controlling genetic variation of the metabolome

**DOI:** 10.3389/fpls.2014.00461

**Published:** 2014-09-11

**Authors:** Bindu Joseph, Susanna Atwell, Jason A. Corwin, Baohua Li, Daniel J. Kliebenstein

**Affiliations:** ^1^Department of Plant Sciences, University of California Davis, Davis, CAUSA; ^2^DynaMo Center of Excellence, University of CopenhagenFrederiksberg, Denmark

**Keywords:** QTL, quantitative genetics, metabolite, glucosinolate, RIL population

## Abstract

A central goal of systems biology is to develop models that are both predictive and accurately describe the biological system. One complexity to this endeavor is that it is possible to develop models that appear predictive even if they use far fewer components than the biological system itself uses for the same process. This problem also occurs in quantitative genetics where it is often possible to describe the variation in a system using fewer genes than are actually variable due to the complications of linkage between causal polymorphisms and population structure. Thus, there is a crucial need to begin an empirical investigation into the true number of components that are used by biological systems to determine a phenotypic outcome. In this study, we use a meta-analysis of directly comparable metabolomics quantitative studies using quantitative trait locus mapping and genome wide association mapping to show that it is currently not possible to estimate how many genetic loci are truly polymorphic within *Arabidopsis thaliana*. Our analysis shows that it would require the analysis of at least a 1000 line bi-parental population to begin to estimate how many polymorphic loci control metabolic variation within *Arabidopsis*. Understanding the base number of loci that are actually involved in determining variation in metabolic systems is fundamental to developing systems models that are truly reflective of how metabolism is modulated within a living organism.

## INTRODUCTION

A central goal of systems biology is to develop models that are both predictive and accurately describe the biological system. Complicating this endeavor is the observation that it is possible to develop highly accurate models that use far fewer components than the biological system itself uses for the same process. The absence of components means that the models typically are not predictive when moving into new areas (phenotypes, environments, species) other than the explicit conditions in which the model was developed. This is because in these different areas there are new and previously unrecognized components that need to be included to make the model accurate. Thus, while it is possible to create highly accurate models, these models ability to be predictive into new untested conditions is frequently hindered. Solving this requires developing a base understanding of how large a true molecular network is within a biological system to ensure that the models are of similar scale.

This reduction conundrum also occurs in quantitative genetics where it is often possible to accurately describe the variation in a system using far fewer genetic loci than may actually be causing the phenotypic variation. This potential arises from the fact that most genetic populations are smaller than required to allow all independent components varying within the population to behave independently (Brem et al., 2005; West et al., 2007; Buckler et al., 2009; Chan et al., 2010b, 2011; Bloom et al., 2013; Albert et al., 2014). This lack of independence arises from the fact that genes are genetically linked upon chromosomes and there has not been sufficient recombination to separate them (Falconer and Mackay, 1996; Mackay, 2001; Manolio et al., 2009). Additionally, most populations do not have sufficient numbers of individuals to fully sample the genetic matrix. For example it would require ∼33000 yeast lines to sample all possible combinations of 15 loci once (Albert et al., 2014). Another factor affecting independence in quantitative genetics is that there may be natural or artificial selection structuring the genome and further decreasing randomness and hence independence (Platt et al., 2010a,b). Thus, there is a crucial need to begin an empirical investigation into the true number of variable causal loci that may be present in any mapping population.

A key approach to study the number of loci causing variation in a phenotype is the use of structured mapping populations. Modeling studies often test how the size of a mapping population and its recombination design impact the ability to find quantitative trait loci (QTLs). However, these modeling studies are typically built on the assumption that existing populations have largely discovered what is available to be discovered within a specific population. This arises because studies often bootstrap the analysis by taking a subset of the population and then testing how many of the final QTLs were found (de Koning et al., 1998; Charmet, 2000; Perretant et al., 2000; Doerge, 2002; West et al., 2007). These analyses always show that only a smaller subset of the lines were necessary to identify what was found in the full population. This is then frequently misinterpreted to mean that all potential QTLs present within this population were found. However, this simply means that the researcher did not need the full population to find what they found. In contrast, this backward bootstrapping has no predictive capacity to provide information on what additional information might have been found if the population had been even larger than tested. Thus there is a need to conduct an empirical analysis of how population size may influence the number of QTLs being detected as a first step to figuring out how many loci may exist within a population or species.

In the report, we conduct a meta-analysis of metabolite QTL mapping studies within simple bi-parental recombinant inbred line (RIL) populations of *Arabidopsis thaliana* to begin studying what is necessary to estimate the number of loci that affect a trait in a species. We show that the different RIL populations have similar genetic architecture suggesting that they can be treated as a randomized sample of the species. Using multiple populations of differing sizes measured for the same phenotypes with the same experimental and statistical approaches, we show that the number of QTL identified increases with population size but that it is currently impossible to tell if this relationship is linear or log-linear. Separating these two models is essential to developing future populations but will require a bi-parental RIL population of at least 1000 lines. Interestingly, the new QTLs identified with the increasing population sizes were not of small effect but instead they were of similar effect to the QTLs found in smaller populations. In contrast, most models assume that as more QTL are found they are of smaller and smaller effect. This means that we may be vastly underestimating the genetic potential present within any single RIL population. If we are underestimating a simple bi-parental population than there is an even larger issue of underestimation with more complex multi-parent or genome wide association (GWA) mapping population. New empirical and modeling studies taking into account these meta-analysis observations will be needed to design optimal mapping populations for future analysis.

## MATERIALS AND METHODS

### METABOLOMICS META-ANALYSIS OF RIL POPULATIONS

We obtained all the QTL mapping data from two previous experiments looking at metabolomics QTLs in the Bay × Sha and Kas × Tsu RIL populations (Loudet et al., 2002; McKay et al., 2008; Rowe et al., 2008; Juenger et al., 2010; Joseph et al., 2013a). These experiments were done in the same growth chamber using the same experimental protocols optimizing the ability to directly compare the results. Additional metabolomics QTL studies in *Arabidopsis* RIL populations were not included either because they used different experimental designs that prevented the ability to compare the results or the appropriate data were not available (Keurentjes et al., 2006; Lisec et al., 2008, 2009; Sulpice et al., 2009; Brotman et al., 2011). All metabolomics were conducted at the University of California Davis metabolome facility following the same published protocols as described in the direct citations for each dataset (Weckwerth et al., 2004; Fiehn et al., 2005, 2008; Rowe et al., 2008; Chan et al., 2010a; Joseph et al., 2013a).

### METABOLOMICS META-ANALYSIS OF GWA IN *Arabidopsis*

To compare the genetic architecture of metabolome variation in RILs with GWA populations, we obtained all the metabolite variation data from a previous GWA analysis of 96 accessions that were done in the same growth chamber using the same experimental protocols (Chan et al., 2010a). These 96 accessions were the same as described in other GWA analysis (Atwell et al., 2010). This allowed us to optimize the comparability of the results.

### GLUCOSINOLATE META-ANALYSIS OF RIL POPULATIONS

For our meta-analysis of glucosinolate QTL analysis, we obtained all QTL mapping data from previous experiments looking at glucosinolate QTLs in the L*er* × Col-0, L*er* × Cvi, Bay × Sha, Da(1)-12 × Ei-2, and Kas × Tsu RIL populations (Kliebenstein et al., 2001b, 2002a,b; Wentzell et al., 2007; Joseph et al., 2013b). The number of QTLs found for each trait were available for all populations but the estimated additive effect per locus was only available for the Bay × Sha, Da(1)-12 × Ei-2, and Kas × Tsu RIL populations (Kliebenstein et al., 2001b, 2002a,b; Wentzell et al., 2007; Joseph et al., 2013b). These QTL studies were all conducted with the same technical platform and similar replication allowing for an optimal comparison of the results (Kliebenstein et al., 2001b, 2002a,b; Wentzell et al., 2007; Joseph et al., 2013b). For all experiments, they were conducted using the same established high-throughput glucosinolate extraction protocol with the same quantification approaches and level of replication (Kliebenstein et al., 2001a,b,c; Reichelt et al., 2002).

### STATISTICAL ANALYSIS

All statistical analysis and visualizations were conducted within the R software (R Development Core Team, 2014).

## RESULTS

### COMPARATIVE METABOLOME HERITABILITY ACROSS POPULATIONS

**FIGURE 1:**
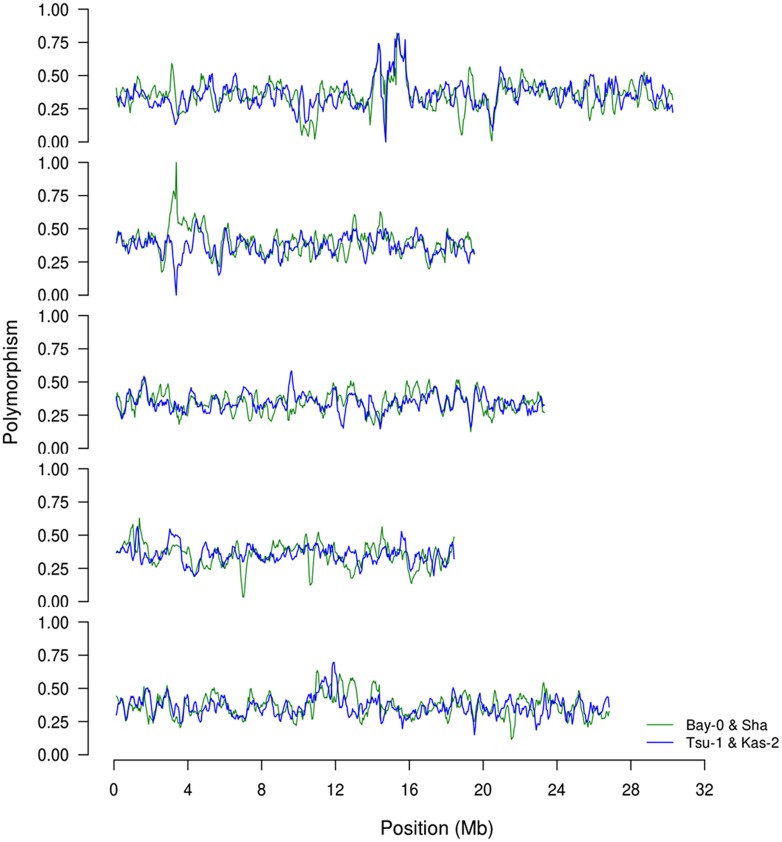
**Comparison of SNP distributions in the recombinant inbred line (RIL) populations.** We obtained previously published single nucleotide polymorphism calls between the four genotypes, Bay, Sha, Kas, and Tsu. Using this data, we estimated the distribution of SNPs on chromosomes 1–5 (top to bottom) based on a sliding window of 250 Kb along the chromosome. The frequency is shown as the fraction of polymorphisms per 100 bp between the two parents for BayxSha (green) and KasxTsu (blue) RIL populations.

**FIGURE 2:**
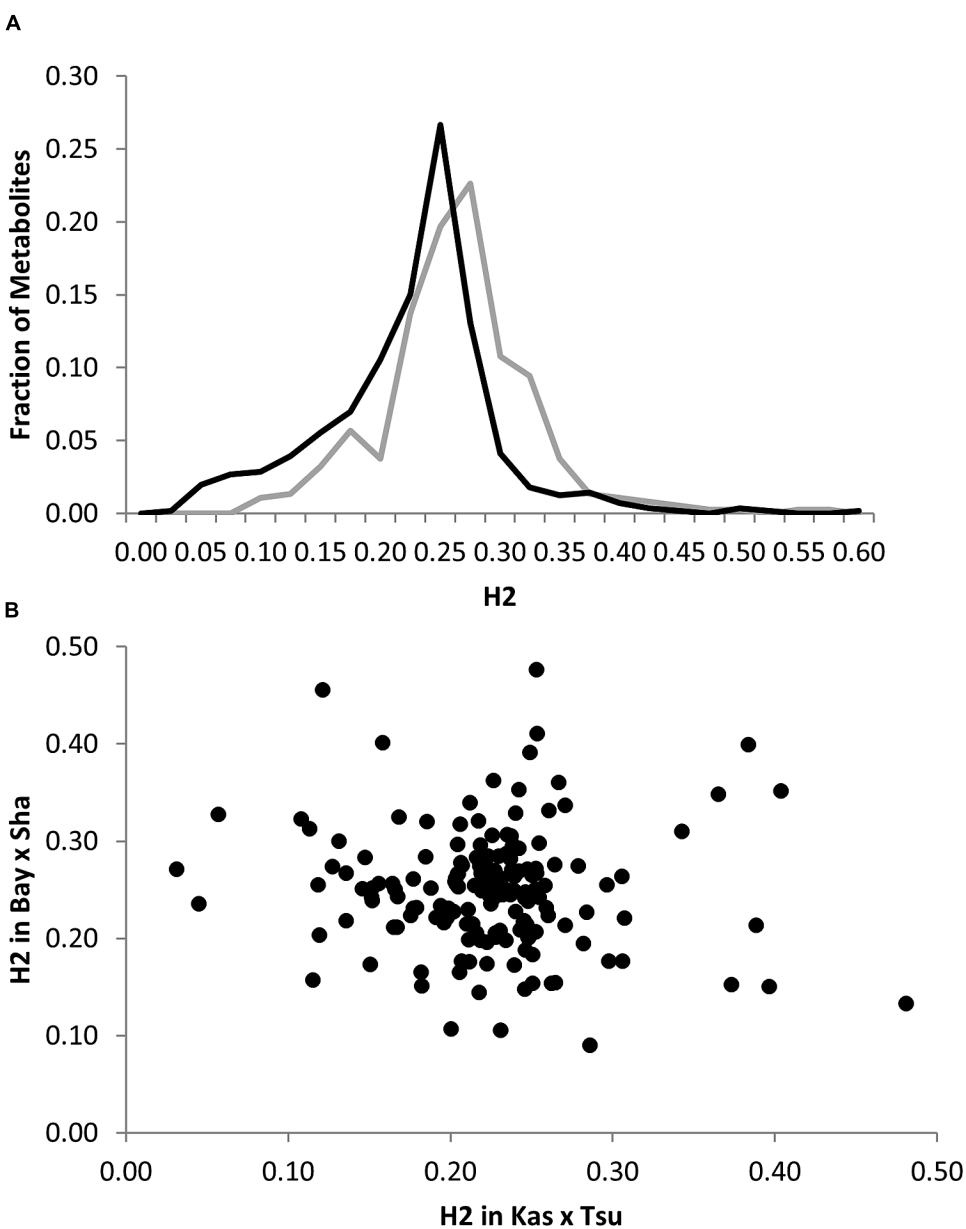
**Comparative metabolite genetics. (A)** Comparison of estimated metabolite heritability’s in the Bay × Sha (gray) and Kas × Tsu (black) RIL populations. **(B)** Scatter plot of heritability for 258 metabolites where both heritability and replicate effect could be estimated in the Bay × Sha and Kas × Tsu RIL populations. No significant correlation was found (Pearson correlation).

To begin investigating how population size and diversity may influence metabolome QTL identification, we compiled data from two metabolomics studies in which the same experimental design, metabolome analysis protocol and growth chambers were used (Rowe et al., 2008; Joseph et al., 2013a). This minimizes any technical or environmental difference between the experiments that could influence the comparison. The two metabolomics QTL studies used RIL populations of different sizes; the Kas × Tsu *A. thaliana* RIL population had 316 lines while the Bay × Sha population had only 210 lines measured (Loudet et al., 2002; McKay et al., 2008; Rowe et al., 2008; Joseph et al., 2013a,b). Further, the two populations are highly diverse with minimal shared regions of high or low polymorphism indicating that we can treat them as a random sampling of potential RILs that may be generated from *Arabidopsis* (**Figure 1**; Atwell et al., 2010). 258 predominantly primary metabolites were detected in the QTL mapping experiments for both populations allowing these metabolites to be used for a direct comparison of the genetics controlling the plant metabolome between these two populations (Rowe et al., 2008; Joseph et al., 2013a). The two populations showed a highly similar heritability distribution for the metabolome variation, 21% for Kas × Tsu and 25% for Bay × Sha (**Figure 2A**; Rowe et al., 2008; Joseph et al., 2013a). In contrast, a direct comparison of the heritability for each specific metabolite in the two populations showed that there was no significant correlation across the metabolites (**Figure 2B**, Pearson correlation, *r*^2^ = 0.04, *P* = NS; **Figure 2B**; Rowe et al., 2008; Joseph et al., 2013a). Thus, while the genetics affecting the metabolome has similar overall heritability in the two populations, this genetic diversity affects different metabolites in the two populations. As such the size of the RIL population did not influence the distribution of heritability’s.

### SHARED STRUCTURE BUT DIFFERENT SPECIFICS OF METABOLOMIC DIVERSITY IN RIL POPULATIONS

**FIGURE 3:**
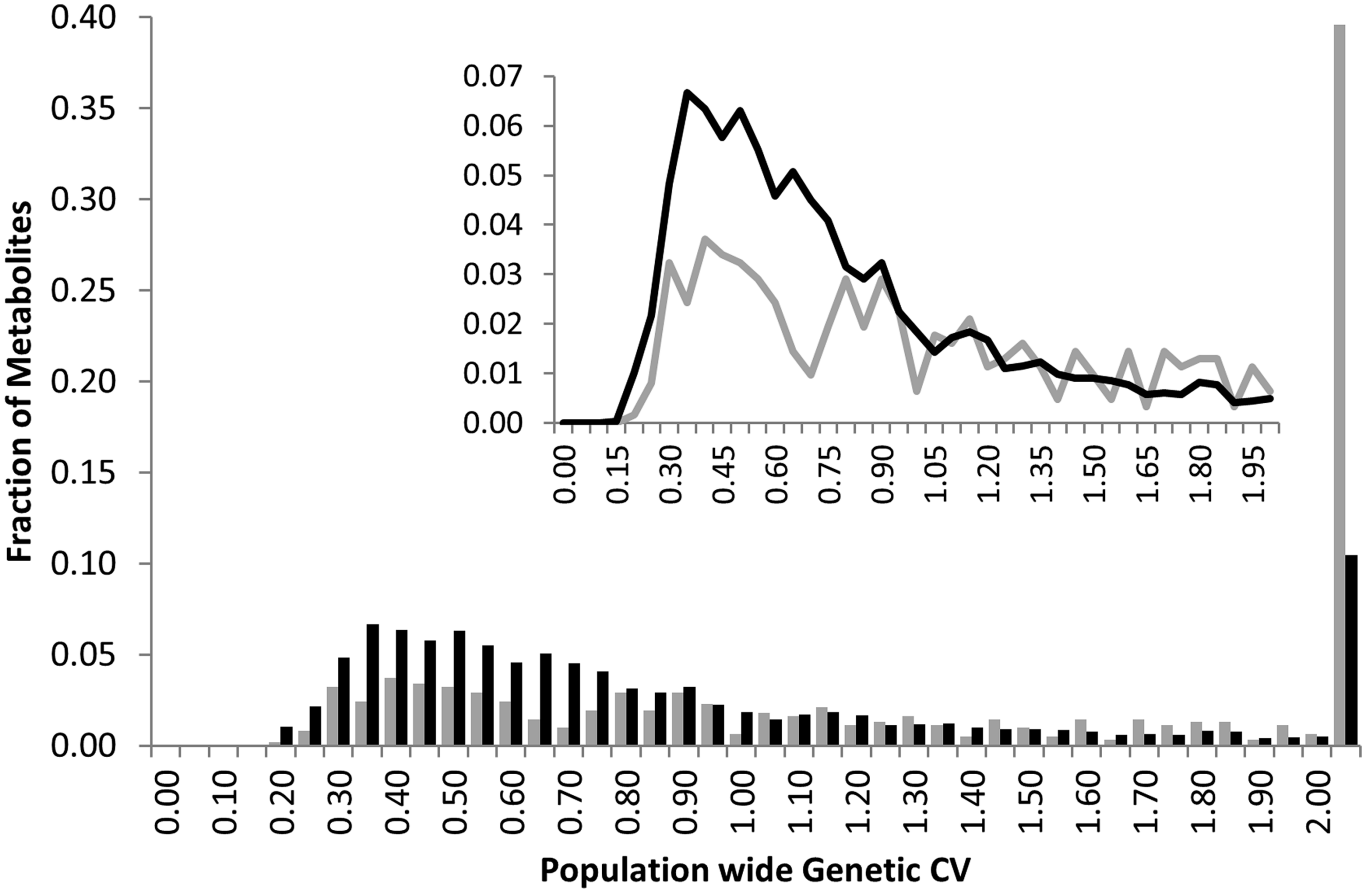
**Comparative metabolite genetics.** Comparison of population CV across the metabolites within the Bay × Sha (gray) and Kas × Tsu (black) RIL populations. Inset shows a zoomed in view of the majority of the distribution.

**FIGURE 4:**
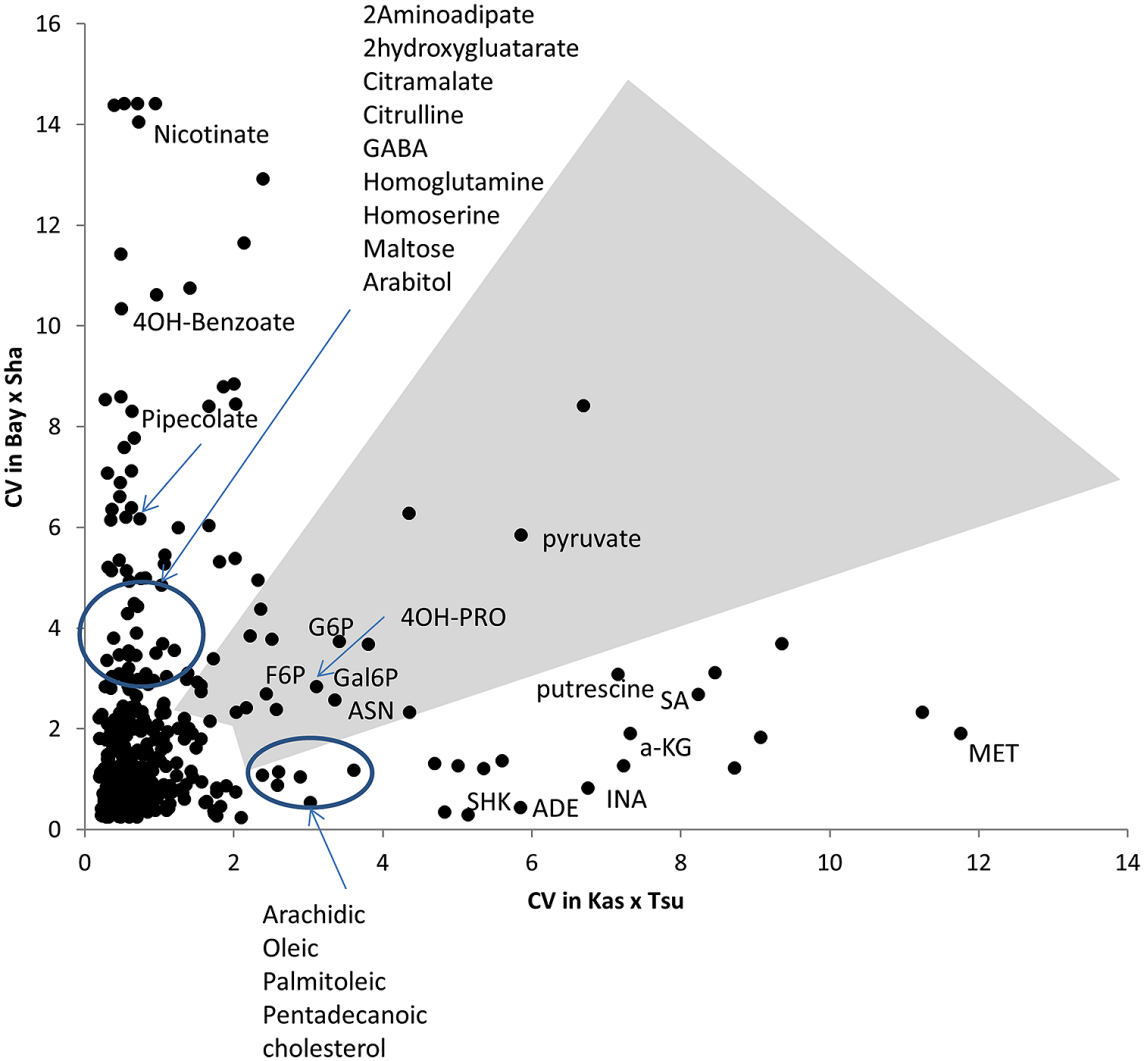
**Different modules of metabolic variation in different populations.** Comparison of metabolite CV across all the RILs in the Bay × Sha and Kas × Tsu populations for 258 metabolites where CV could be estimated in both populations and that showed significant heritability’s in both populations. The gray polygon shows metabolites with elevated phenotypic variation in both populations. Below the polygon are metabolites with high variation only in Kas × Tsu while to the left are metabolites that show elevated variation in only Bay × Sha. Known metabolites are labeled while unknown metabolites are unlabeled. Elevated variance is defined as being in the top 10th percentile of metabolites within a population.

Heritability showed that the overall genetic architecture affecting the metabolome is similar between the two populations but that the affected metabolites are different. We further tested this using the genetic variance present in each metabolite across the RILs. To do this, we compared the genetic CV for all 258 metabolites across the two populations. As with heritability, the overall distribution of metabolic variance was very similar between the two populations (**Figure 3**). The larger Kas × Tsu population was slightly skewed toward metabolites with larger population variance while the smaller Bay × Sha population had a slight enrichment in metabolites with lower genetic variance (**Figure 3**; Rowe et al., 2008; Joseph et al., 2013a). As with heritability, plotting the genetic CV of the 258 metabolites detected in both populations showed no correlation indicating that different specific metabolites are affected by this similar genetic architecture (**Figure 4**, Pearson correlation).

Within the Bay × Sha population the highly variable metabolites were enriched in sugars and amine metabolic processes (**Figure 4**). In contrast, the metabolites specifically variable in the Kas × Tsu population are more associated with stress responses like Shikimate, putrescine, SA, and isonicotinic acid or lipid metabolism (**Figure 4**). Interestingly, the known compounds that displayed elevated genetic variance in both populations are key energy balance compounds like asparagine, pyruvate, glucose-6-phosphate, fructose-6-phosphate and galactose-6-phosphate (**Figure 4**). Typically, these central energy flux components are considered constrained in their function which should limit their diversity but this does not appear to be the case in *Arabidopsis*. The lack of correlation amongst specific metabolites for heritability or genetic CV between the two populations argues that each population has specific genetic polymorphisms that alter distinct metabolites in each population. These genetic polymorphisms are largely not shared between the two populations (**Figure 1**). Thus, while the specific genetic variation in each RIL population affects different metabolites, the overall genetic architecture (the distribution of heritabilities and CV) of each population is similar. The fact that the overall genetic architecture of the RIL populations is comparable suggests that we can treat diverse RIL populations in *Arabidopsis* as random sample of the potential genetic diversity within the species.

### COMPARATIVE METABOLOME GENETICS ACROSS POPULATIONS

**FIGURE 5:**
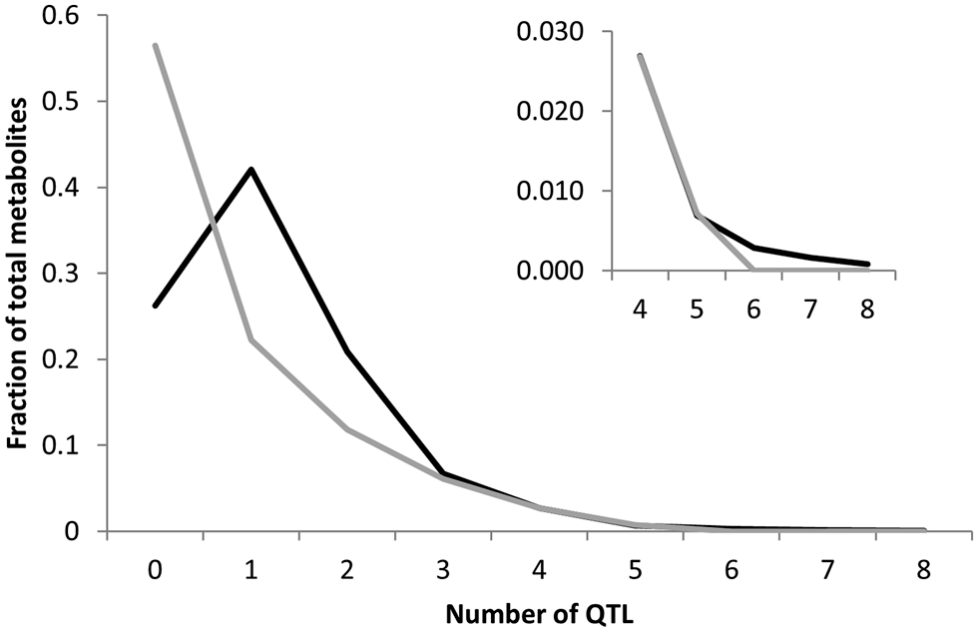
**Comparison of QTL detection across populations.** Shown is the frequency of metabolites that detected a given number of nuclear genome Quantitative trait loci (QTLs) in the Bay × Sha RIL population (gray) and the Kas × Tsu RIL population. Inset shows a magnification of the *X* axis in the four to eight QTL region.

**FIGURE 6:**
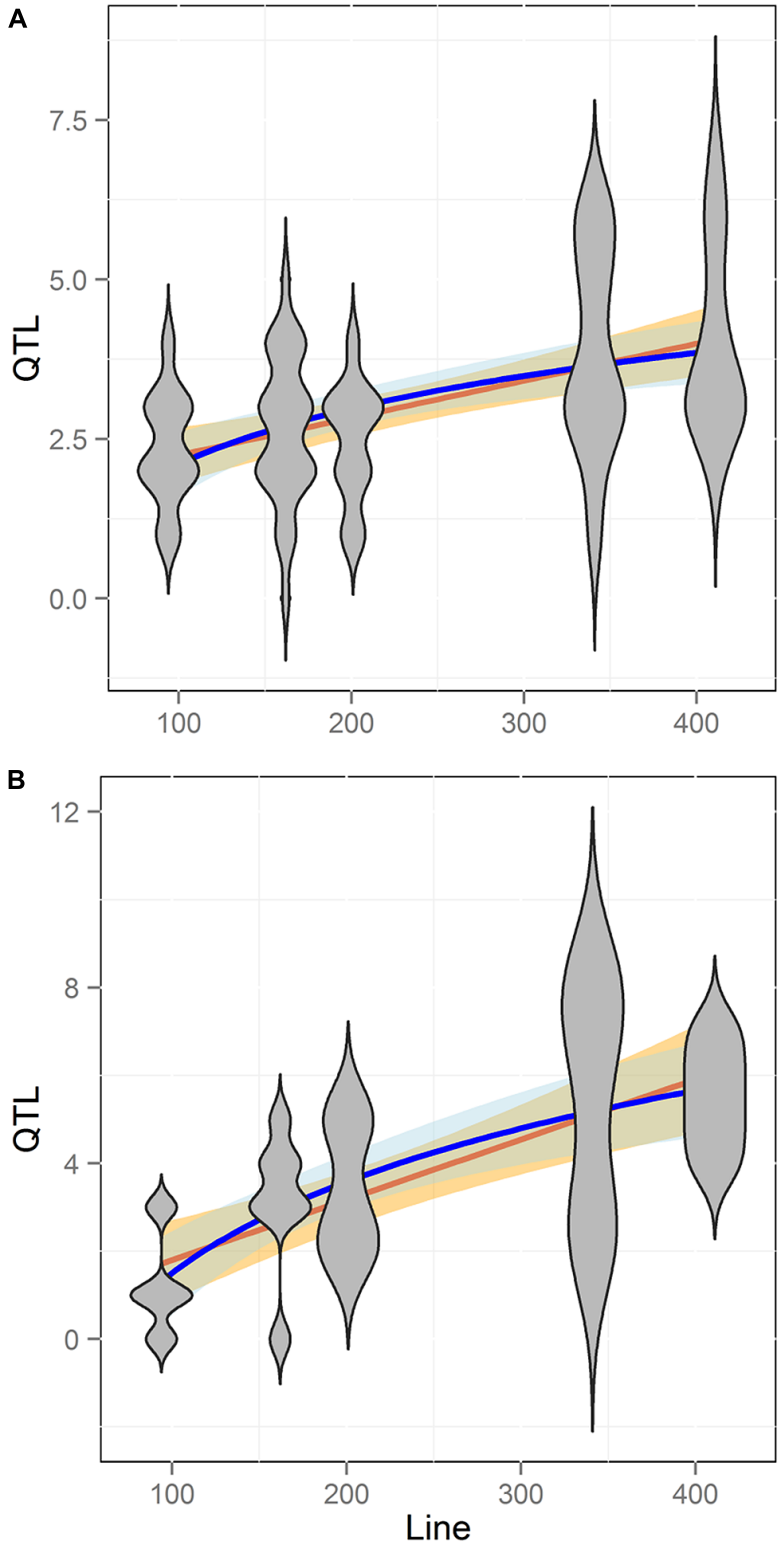
**Comparison of QTL detection across populations.** Shown is the number of QTL identified across five different RIL populations of *Arabidopsis* with different line numbers. Blue shows the estimated log-linear relationship of QTL # identified per Line # while Orange shows the estimated linear relationship. SE of the estimated relationships are shown in filled color. Slopes and confidence intervals were obtained using Pearson correlation of either the linear or log-adjusted data. Plots are shown in linear scales for comparison purposes.** (A)** Aliphatic glucosinolate traits **(B)** Indolic glucosinolate traits.

To begin testing how an increase in the number of lines between the two populations increases the ability to identify QTLs, we compared the number of QTLs identified for the 258 common metabolites. For both populations we had previously used the same composite interval mapping (CIM) approach to identify and call significant QTLs. In combination with the similar range of heritability’s and genetic variances, this identical statistical approach allows us to conduct a direct comparison where the only major difference in the two populations is line number (Jansen, 1994; Zeng et al., 1999a; Broman et al., 2003; Rowe et al., 2008; Joseph et al., 2013a). There was 54% more QTLs per metabolite detected in the 316 line Kas × Tsu population than for the 211 line Bay × Sha population (**Figure 5**). The Kas × Tsu population had an average of 1.22 ± 0.02 QTLs per metabolite in comparison to 0.79 ± 0.1 QTLs per metabolite found in the Bay × Sha RIL (**Figure 6**; Avg ± S.E.). The main difference between the two populations was the number of metabolites with at least one detected QTL; Kas × Tsu had a QTL detected for 75% of metabolites while for Bay × Sha this was only 44%. There was also an increase in the number of metabolites with two or more QTLs (**Figure 5**). Interestingly, 316 lines represents about a 50% increase in the number of lines which is similar to the 50% increase in QTL detected suggesting the potential for a linear relationship between the number of lines present in a population to the number of metabolite QTL detected.

### RIL POPULATION SIZE AND QTL DETECTION USING TARGETED METABOLITE ANALYSIS

To better test how RIL population size influences the ability to identify QTLs we obtained data on QTL mapping for aliphatic and indolic glucosinolate accumulation within five different *Arabidopsis* populations (Lister and Dean, 1993; Alonso-Blanco et al., 1998; Kliebenstein et al., 2001b, 2002a,b; Loudet et al., 2002; Pfalz et al., 2007; Wentzell et al., 2007; McKay et al., 2008; Joseph et al., 2013b). These RIL populations differ in size from 100 to 411 lines and the glucosinolates were measured in the same tissue with similar replication using the same technical platform. Additionally, the glucosinolate QTL mapping was done with the same algorithm for all experiments (Kliebenstein et al., 2001b, 2002a,b; Wentzell et al., 2007; Joseph et al., 2013b). This allows us to conduct a direct comparison of QTL detection where the major difference is solely due to differences in the populations. Comparing the number of QTL identified to the number of lines in each population showed that QTL identification significantly increased with population size (**Figure 6**; Pearson Correlation, *P* < 0.001). This increase in QTL identification with population size was found for both aliphatic and indolic glucosinolates (**Figure 6**; Pearson Correlation, *P* < 0.001 for both). Within these populations, the aliphatic and indolic glucosinolates are controlled by different causal loci suggesting that they are behaving as independent measures of the relationship between power to identify QTL and population size within these populations (Kliebenstein et al., 2001b, 2002a,b; Pfalz et al., 2007; Wentzell et al., 2007; Joseph et al., 2013b).

**FIGURE 7:**
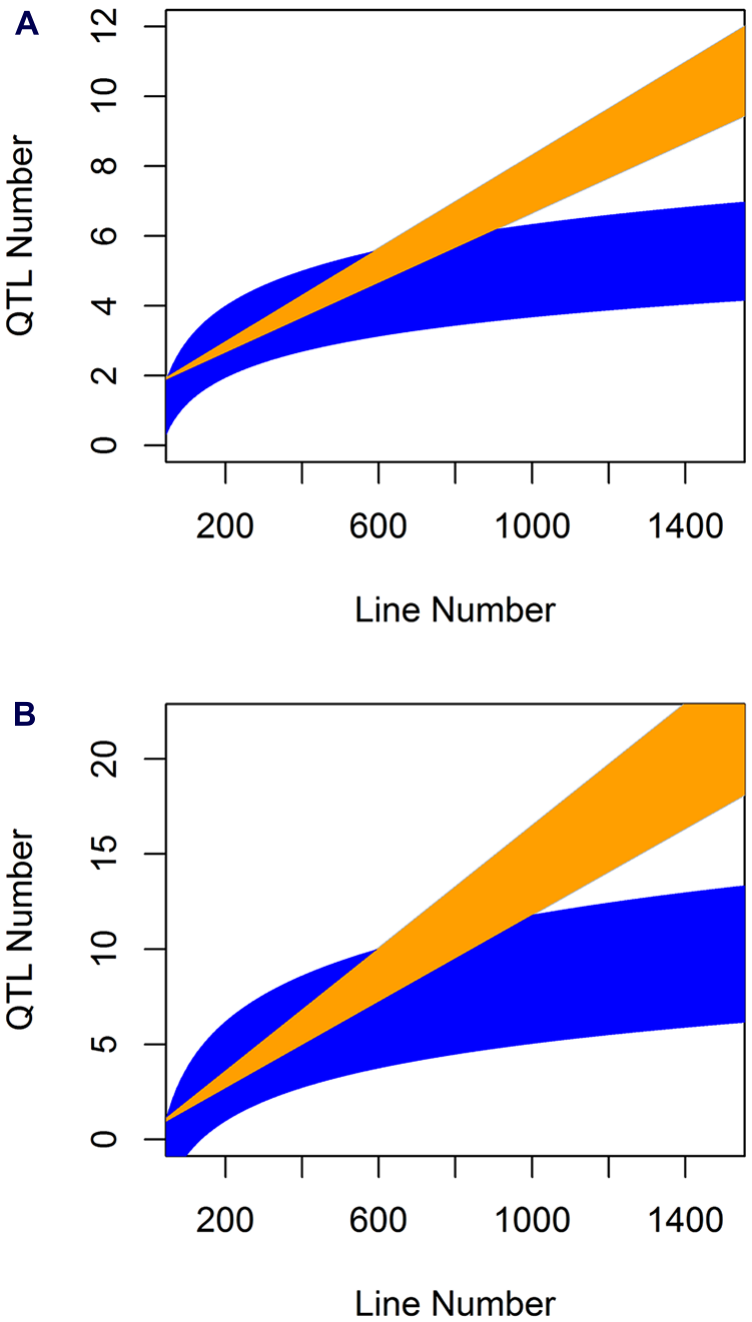
**Comparative prediction of QTL detection across population size.** The slope and 95% confidence interval in the slope for both the linear and log-linear regression of QTL number by line number was obtained for the aliphatic **(A)** and indolic **(B)** glucosinolates across five populations where they were measured with the same experimental design and technical protocol. These estimated slopes were then use to plot the predicted QTL number detected per populations of a given line under the linear and log-linear predictions. Yellow shows the linear prediction and blue the log-linear prediction.

When analyzing the relationship between QTL number found and population size for the glucosinolate phenotypes we found a log-linear relationship between the two parameters as has often been found in modeling studies (**Figure 6**; Pearson correlation using log adjusted values, *P* < 0.001 for both; Falconer and Mackay, 1996; Mackay, 2001; Doerge, 2002; Stich et al., 2007; West et al., 2007; McMullen et al., 2009; Klasen et al., 2012). However, a linear regression was also an equal statistical fit to the data (**Figure 6**). Thus, the existing data cannot differentiate between a linear and log-linear relationship of QTL number detected to population size. This is even though the large population is considered to be sufficient to fully sample the potential QTL in a population (**Figure 6**; Falconer and Mackay, 1996; Mackay, 2001; Doerge, 2002; Stich et al., 2007; West et al., 2007; McMullen et al., 2009; Klasen et al., 2012). Using the regression estimates we projected the linear and log-linear regression models with their 95% confidence intervals to larger population sizes to test what population size would be required to differentiate between these two different regressions (**Figure 7**). Even though both the aliphatic and indolic glucosinolates had different specific regression estimates the two traits generated the same estimate that it would require minimally between 950 and 1000 individuals in a single bi-parental RIL population to test which regression model more accurately approximates the relationship between population size and the power to identify new QTLs (**Figure 7**).

### RIL POPULATION SIZE AND QTL EFFECT SIZE

**FIGURE 8:**
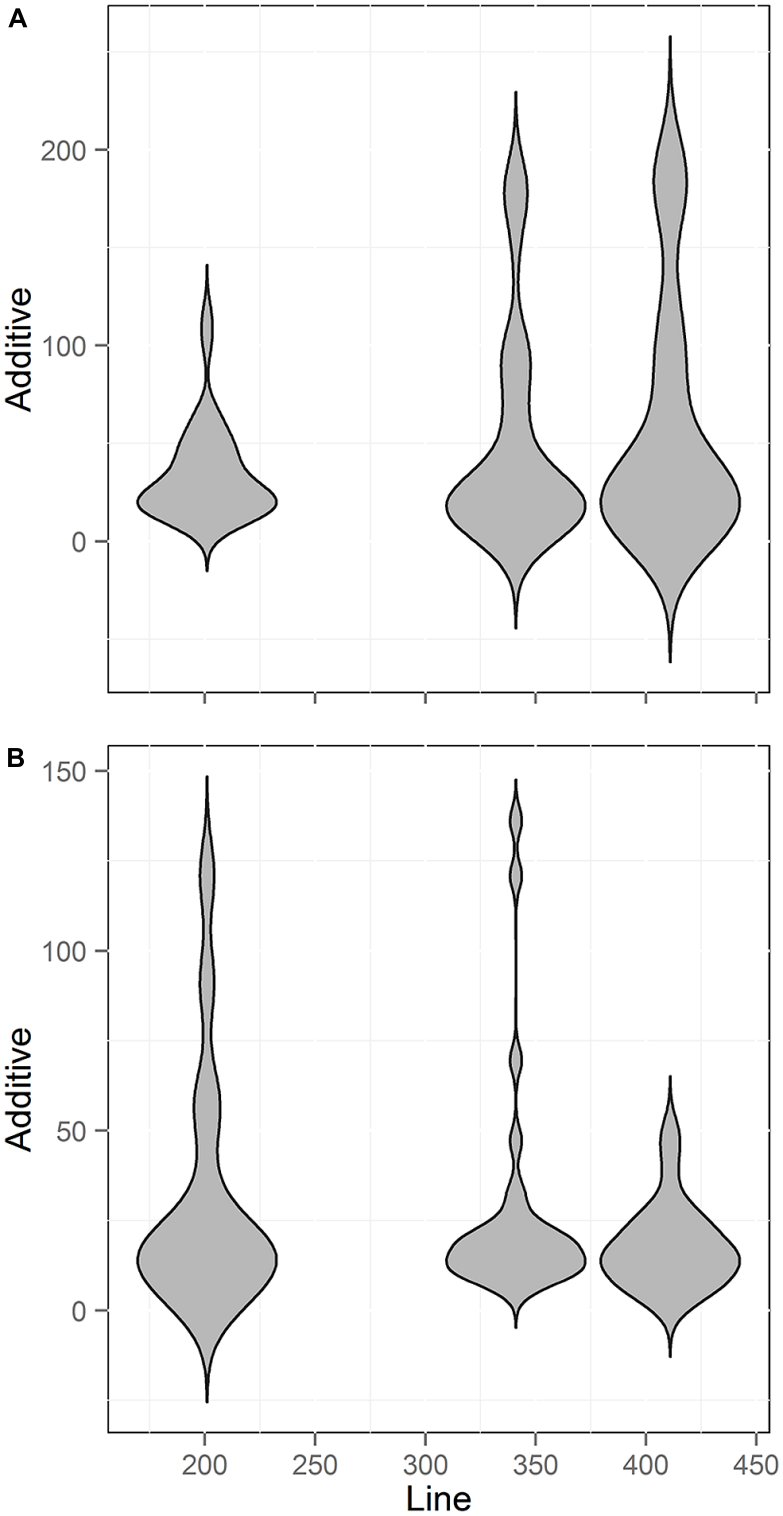
**Comparison of QTL Effect sizes by population size.** Shown is the number of QTL identified across three different RIL populations of *Arabidopsis* with different line numbers. Two smaller populations could not be included because estimates of additive effects were not published. **(A)** Aliphatic glucosinolate traits **(B)** Indolic glucosinolate traits.

A common assumption in QTL mapping is that small RIL populations will identify larger effect loci and the larger populations simply add smaller effect loci (Beavis, 1994, 1998; Zu, 2003). Using our meta-analysis data, we tested if there was a relationship between population size and the additive effect of the QTLs found. We chose to focus on additive effect rather than percent of total variance (*r*^2^) explained because the additive effect of an individual locus is not dependent upon the total population variance. In contrast, the *r*^2^ per locus is determined both by the effect of the locus and the total population variance (Beavis, 1994, 1998; Falconer and Mackay, 1996; Mackay, 2001; Zu, 2003). Thus, additive effect allows for more independence when comparing across populations. We thus compared line number against the additive effect size of each QTL found across three of the populations. Two of the five populations did not have the estimated additive effect size for the QTLs and were not used. Unexpectedly, this comparison showed no statistically significant relationship between population size and the additive effect of the identified QTLs for either the aliphatic or indolic glucosinolates (**Figure 8**; Pearson and Spearman Rank correlation tests). Thus, the new QTL found as population size increased were not of smaller effect but instead of similar effect size to those found with smaller population sizes.

### COMPARATIVE METABOLIC DIVERSITY BETWEEN RIL AND GWA POPULATIONS

**FIGURE 9:**
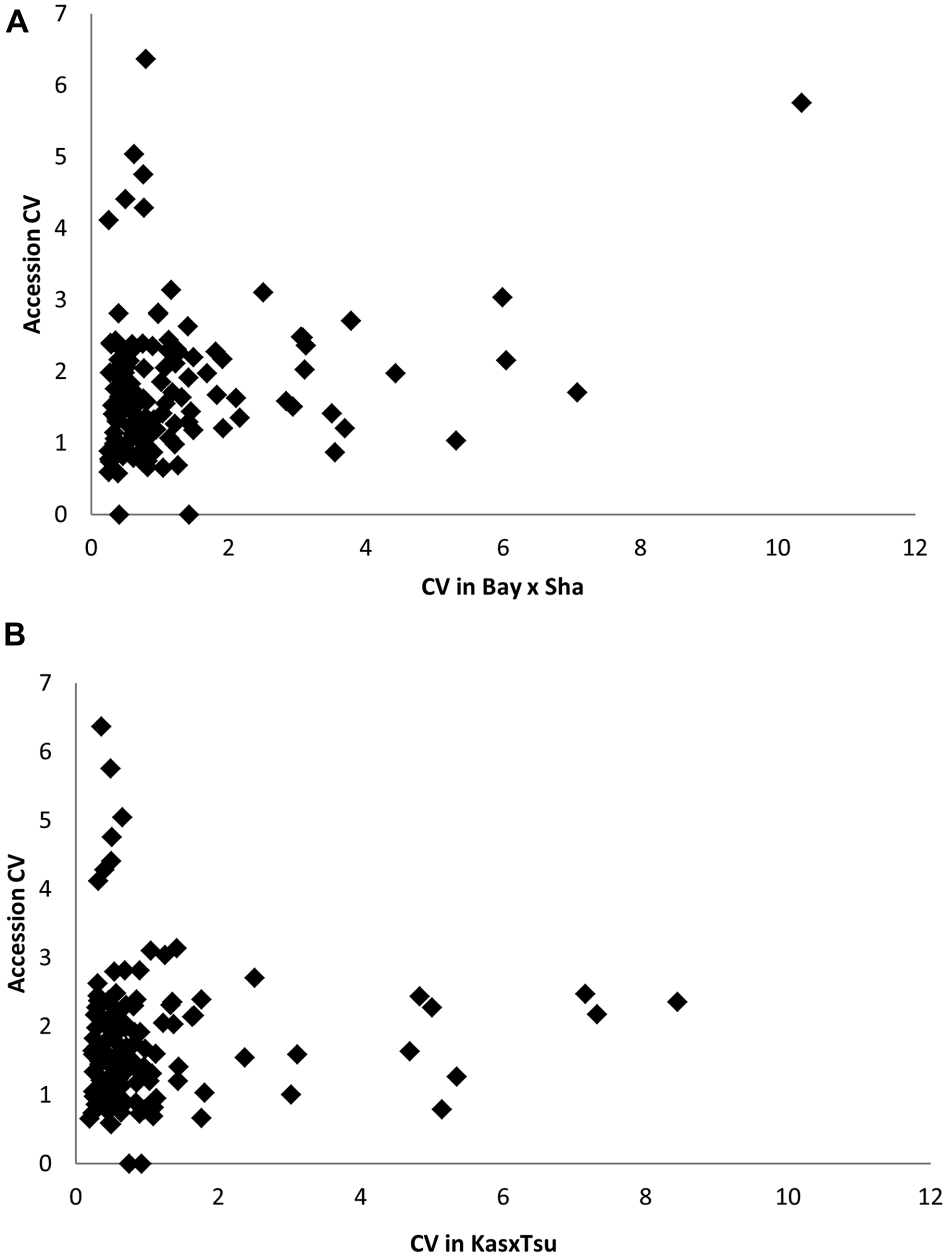
**Comparative phenotype diversity in 96 *Arabidopsis* accessions to RIL populations. (A)** Comparison of Genetic CV for 135 metabolites measured in the Bay × Sha RILs and96 *Arabidopsis* accessions. **(B)** Comparison of Genetic CV for 135 metabolites measured in the Kas × Tsu RILs and96 *Arabidopsis* accessions.

A common concern affecting RIL populations is that there are only two alleles per locus and thus might be limited in their genetic variation relative to species-wide diversity (Kim et al., 2007; Nordborg and Weigel, 2008). A proposed solution to this limitation is the use of unstructured GWA mapping populations that sample greater genetic diversity (Atwell et al., 2010; Chan et al., 2010a, 2011). This bi-allelic aspect of RIL populations is suggested to constrain RIL populations to simply sampling a subset of phenotypic variation in the more genetically diverse unstructured GWA populations. To assess how this bi-allelic structure may or may not constrain a RIL population, we compared the metabolomic analysis of two RIL populations with a GWA population using 135 metabolites detected in all three experiments (Rowe et al., 2008; Atwell et al., 2010; Chan et al., 2010a). These 135 metabolites included most of the known primary compounds (Rowe et al., 2008; Atwell et al., 2010; Chan et al., 2010a). Using the available data, we determined genetic CV for all 135 metabolites measured in each of the populations (Rowe et al., 2008; Atwell et al., 2010; Chan et al., 2010a; **Figure 9**). Comparing the genetic CV showed that the RIL populations could capture a majority of the genetic variance controlling metabolite variation within *Arabidopsis* (**Figure 9**). Additionally, both RIL populations identified variance not present in the accessions as they had at least 14 metabolites showing twice the genetic variance found in the accessions (**Figure 9**). In contrast, there were only five metabolites showing elevated variation in the accessions that was not captured in the two RIL populations (oxoproline, glycerol and three unknowns; **Figure 9**). Thus, while RILs have lower genetic diversity at individual loci than the accessions, this does not limit the associated phenotypic diversity.

## DISCUSSION

### ESTIMATING THE NUMBER OF QTLs PER BI-PARENTAL POPULATION

Obtaining an accurate estimate of how many QTLs exist within a single population is a key parameter for any quantitative modeling study. However, there is no empirical understanding for how this parameter, QTLs per population, should be set within modeling studies. This leads to a massive range of values in these modeling studies complicating any true comparison between these modeling studies (Zeng et al., 1999b; Otto and Jones, 2000; Xu, 2003; Slate, 2013; Guo et al., 2014). Even the analysis of individual large populations reporting large numbers of loci controlling nearly all of the heritability are likely under-estimates due to insufficient recombination between loci (McMullen et al., 2009; Huang et al., 2010; Kump et al., 2011; Tian et al., 2011; Bloom et al., 2013; Albert et al., 2014). In addition to modeling studies, understanding how many QTLs exist within a single population is fundamental to knowing how to design future mapping populations especially with the interest in multi-parent populations (Kover et al., 2009; McMullen et al., 2009). To date, these populations have been modeled and structured based on the assumption that existing bi-parental populations have identified the majority of identifiable QTLs in those populations (Kover et al., 2009; McMullen et al., 2009).

Thus, we conducted a meta-analysis of existing metabolite QTL mapping within simple bi-parental *Arabidopsis* populations to begin an empirical assessment of how many QTLs are present in a given mapping population. As expected, including more lines in a bi-parental did lead to more identified QTLs. However, the relationship between line number and QTL number equally fit a linear and log-linear relationship even with 411 lines (**Figure 6**). Extending these linear and log-linear relationships showed that it would require 950 or more lines for even a simple bi-parental population before we could tell which relationship accurately describes the true number of metabolite QTL per population (**Figure 7**). Accurately testing which model best describes QTL number per a bi-parental population is essential given that most multi-parent populations are stopping at 1000 lines under the assumption that this is sufficient to identify all the QTLs (Flint et al., 2005; Kover et al., 2009). However, our meta-analysis suggests that 1000 lines are only just sufficient in a bi-parental population much less a population with multiple alleles per locus. This indicates that at least one bi-parental population of more than 1000 lines should be developed in *Arabidopsis* to tests if the QTL identification power of a population is a linear or log-linear relationship to its size.

The above analysis is focused on metabolite related QTLs and as such may not be broadly reflective of all traits. For example, expression linked traits show higher average heritability than the metabolomic traits but lower heritability than the glucosinolate related traits used in this study (Keurentjes et al., 2007; West et al., 2007; Rowe et al., 2008; Kliebenstein, 2009; Jimenez-Gomez et al., 2011; Joseph et al., 2013a). Most physiological and defense related phenotypes have heritability’s similar to these metabolomic results within these populations (Joseph et al., 2013b). Given the potential for different genetic architectures in each trait, it argues that there needs to be a broader effort to test if the if the link between QTL identification power and population size is affected by the phenotype being studied.

### QTL EFFECT SIZE DISTRIBUTION

In contrast to most quantitative theory, our analysis found that larger population sizes identified QTLs with a similar distribution of effect sizes as smaller populations (Beavis, 1994, 1998). A possible explanation for how the new QTLs found with increasing line numbers is that there might be a significant confounding issue of linked QTLs in most existing populations (Yamamoto et al., 2014). If two QTLs were linked and had opposing effects there is a high likelihood that in small populations the region would be missed. This is because the two QTLs effects would cancel each other out and the lack of recombination would not allow either locus to be detected. Thus, when these loci are identified in larger populations they could have effect sizes similar to QTLs that are not linked. This linkage of opposing effects has previously been found using larger *Arabidopsis* populations (Wentzell et al., 2007; Rowe and Kliebenstein, 2008; Rowe et al., 2008). Another possibility is that if two QTLs are linked with a similar direction of effects, they might be identified as a single locus in smaller populations but with an inordinately large effect size (Yamamoto et al., 2014). Then upon increasing the number of lines, the two loci would separate into two QTLs of smaller effect than the original locus (Hansen et al., 2007). Combinations of loci with both similar and opposing effects have been found when conducting fine-scale dissection of *Arabidopsis* QTLs (Kroymann and Mitchell-Olds, 2005; Wentzell et al., 2008). Thus, the base assumption that increasing the population sizewill solely identify QTLs of smaller effect size is not supported by this empirical meta-analysis.

## CONCLUSION

Our meta-analysis of QTL analysis using metabolite phenotypes across multiple *Arabidopsis* RIL populations shows that it is not possible to accurately estimate how many QTLs are present in a single population with two alleles per locus. This is in contrast to the myriad of modeling studies that make explicit assumptions about this variable. Future work will be required to extend these populations to larger sizes to provide a direct and empirical estimate of how many QTLs may exist in a population. Conducting these experiments will then provide a more firm foundation for extensions of similar estimates into multi-parent populations and further into the entire species. It is only then that we will have a true view of how many naturally variable genes causally affect variation within the plant metabolome.

## Conflict of Interest Statement

The authors declare that the research was conducted in the absence of any commercial or financial relationships that could be construed as a potential conflict of interest.
